# Multi-label classification of fundus images based on graph convolutional network

**DOI:** 10.1186/s12911-021-01424-x

**Published:** 2021-07-30

**Authors:** Yinlin Cheng, Mengnan Ma, Xingyu Li, Yi Zhou

**Affiliations:** 1 School of Biomedical Engineering, Sun Yat-sen University, No. 132 Waihuan East Road, Guangzhou, 510006 China; 2 Department of Medical Informatics, Zhongshan School of Medicine, Sun Yat-sen University, No. 74 Zhongshan 2nd Road, Guangzhou, 510080 China; 3 Zhongshan School of Medicine, Sun Yat-sen University, No.74 Zhongshan 2nd Road, Guangzhou, 510080 China; 4 Key Laboratory of Tropical Disease Control (Sun Yat-sen University), Ministry of Education, No. 74 Zhongshan 2nd Road, Guangzhou, 510080 China

**Keywords:** Diabetic retinopathy, Fundus images, GCN, Multi-label

## Abstract

**Background:**

Diabetic Retinopathy (DR) is the most common and serious microvascular complication in the diabetic population. Using computer-aided diagnosis from the fundus images has become a method of detecting retinal diseases, but the detection of multiple lesions is still a difficult point in current research.

**Methods:**

This study proposed a multi-label classification method based on the graph convolutional network (GCN), so as to detect 8 types of fundus lesions in color fundus images. We collected 7459 fundus images (1887 left eyes, 1966 right eyes) from 2282 patients (1283 women, 999 men), and labeled 8 types of lesions, laser scars, drusen, cup disc ratio ($C/D>0.6$), hemorrhages, retinal arteriosclerosis, microaneurysms, hard exudates and soft exudates. We constructed a specialized corpus of the related fundus lesions. A multi-label classification algorithm for fundus images was proposed based on the corpus, and the collected data were trained.

**Results:**

The average overall F1 Score (OF1) and the average per-class F1 Score (CF1) of the model were 0.808 and 0.792 respectively. The area under the ROC curve (AUC) of our proposed model reached 0.986, 0.954, 0.946, 0.957, 0.952, 0.889, 0.937 and 0.926 for detecting laser scars, drusen, cup disc ratio, hemorrhages, retinal arteriosclerosis, microaneurysms, hard exudates and soft exudates, respectively.

**Conclusions:**

Our results demonstrated that our proposed model can detect a variety of lesions in the color images of the fundus, which lays a foundation for assisting doctors in diagnosis and makes it possible to carry out rapid and efficient large-scale screening of fundus lesions.

## Background

Diabetic Retinopathy (DR) is the most common and serious microvascular complication in the diabetic population. And it has become the first blinding factor for working-age people worldwide [1, 2]. Timely screening and treatment of DR have been shown to reduce blindness [3]. In order to reduce the socio-economic burden of vision loss caused by retinal diseases, more accurate early screening procedures are needed in high-risk groups. Common DR diagnostic methods are fundus photography of the retinal and fluorescein fundus angiography (FFA). Fundus photography of the retinal can diagnose patients quickly, but its accuracy depends largely on the experience of the physician. FFA can clearly reflect the pathology of the blood vessels in the retina fundus, but it takes a long time and may cause a variety of adverse reactions [4, 5]. Therefore, compared with FFA, fundus photography of the retinal is often used in the researches of fundus disease diagnosis algorithms, which makes it possible to carry out large-scale fundus disease screening rapidly and efficiently, and is conducive to the early detection and treatment of DR patients [6–14].

The common DR diagnosis method is the retinal fundus images detection. But the fundus images detection is time-consuming and its accuracy also depends on the doctor’s experience. With the development of artificial intelligence analysis algorithms for medical images, more and more people have established a series of automatic diagnosis algorithms for DR. According to the purpose of detection, fundus image diagnosis algorithms can be divided into two categories.

One is to detect the fundus image based on the international diabetic retinopathy grade to determine the severity of the patient’s DR. International diabetic retinopathy is divided into 5 grades: normal (no DR), mild non-proliferative DR (mild NPDR), moderate non-proliferative DR (moderate NPDR), severe non-proliferative DR (severe NPDR) and proliferative DR (PDR). In 2008, J. Nayak’s team [6] used the traditional image feature extraction method to extract the features of the fundus images, and then inputted these features into the artificial neural network (ANN). They automatically classified 140 patients for their NPDR and PDR stages of DR. The final accuracy, sensitivity and specificity were 93%, 90% and 100% respectively. In 2016, Pratt’s team [7] proposed a multi-layer convolutional neural network, which achieved 75% accuracy and 30% sensitivity on the dataset of diabetic retinopathy provided by Kaggle. In the same year, Google built a prediction model based on Inception-v3, and achieved an AUC of 0.991, a sensitivity of 90.3%, and a specificity of 98.1% on the EyePACS validation dataset. At the same time, the test classification of Google’s model on the Messidor-2 dataset achieved an AUC of 0.990, a sensitivity of 0.870, and a specificity of 0.985 [8]. In 2018, Google improved its algorithm proposed in 2016. They increased the training dataset, enlarged the input size of the model, and improved the model architecture. The model predicted the 5-level grades of international diabetic retinopathy and achieved an AUC of 0.986, a sensitivity of 0.971, a specificity of 0.923 on the EyePACS validation dataset [9]. In 2019, Xu et al. [10] evaluated DR based on red lesions and bright lesions of fundus images. The model was tested with 19,904 fundus images. The AUC values of the model were PDR, 0.80; severe NPDR, 0.80; moderate NPDR, 0.77; and mild NPDR, 0.78.

The other is to detect the fundus images based on common lesions, and to use more accurate descriptions of the lesions in the fundus image. In 2010, García’s team [15] used radial basis function (RBF) to detect red lesions in the fundus images. The model they proposed was tested on 65 images and obtained an average sensitivity of 100%, an average specificity of 56.00% and an average accuracy of 83.08% on the image scale. In 2017, Tan’s team [16] proposed a ten layers convolutional neural networks (CNN) for DR lesion detection on the pixel scale. The model achieved a sensitivity of 0.8758 and 0.7158 for exudates and dark lesions on 30,275,903 effective points of the CLEOPATRA database. It also achieved a sensitivity of 0.6257 and 0.4606 for hemorrhages and micro-aneurysms. In 2018, Khojasteh et al. [11] used a ten layers CNN to extract the deep features of the fundus image based on patch, and detected exudates, hemorrhages and microaneurysms, the results of the proposed approach shown overall accuracy for DIARETDB1 was 97.3% and 86.6% for e-Ophtha. In 2020, Pan’s team [17] proposed a multi-label classification model for automatic analysis of fundus fluorescein angiography. The area under the curve (AUC) of the model reached 0.8703, 0.9435, 0.9647, and 0.9653 for detecting non-perfusion regions (NP), microaneurysms, leakages, and laser scars respectively on the dataset containing 4067 FFA images.

Common lesions related to DR in fundus images include microaneurysms, macular edema, hard lipid exudates, cotton wool-like soft exudates, etc. In real clinical diagnosis, these lesions may co-exist in fundus images. The simple solution to this multi-lesion detection is to treat each lesion independently, and to turn the multi-label classification problem into multiple binary classification problem. However, these methods ignore the potential relationship between lesions, so they are limited in nature.

In order to detect the multiple lesion labels in fundus images at the same time and to have a solid consideration about the complex topology of the lesion labels, we perform implicit modeling based on the correlation between the labels of graph convolutional network and build a fundus images multi-label classification model. First of all, we collecte enough papers related to fundus lesions through keywords searching from China National Knowledge Infrastructure (CNKI) website [18] to construct a corpus. After that, we use the method of Global Vectors for Word Representation (Glove) to build a word vector model based on the corpus, and use the word vector model to construct a directed graph of the target label. Then we use the GCN network to model the label dependency. In the end, we use the convolutional neural network to extract image features and combined the output of the GCN network to simultaneously detect lesions. These lesions include laser scars, drusen, cup disc ratio ($C/D>0.6$), hemorrhages, retinal arteriosclerosis, microaneurysms, hard exudates, soft exudates and so on.

## Methods

### Proposed model

**Fig. 1:**
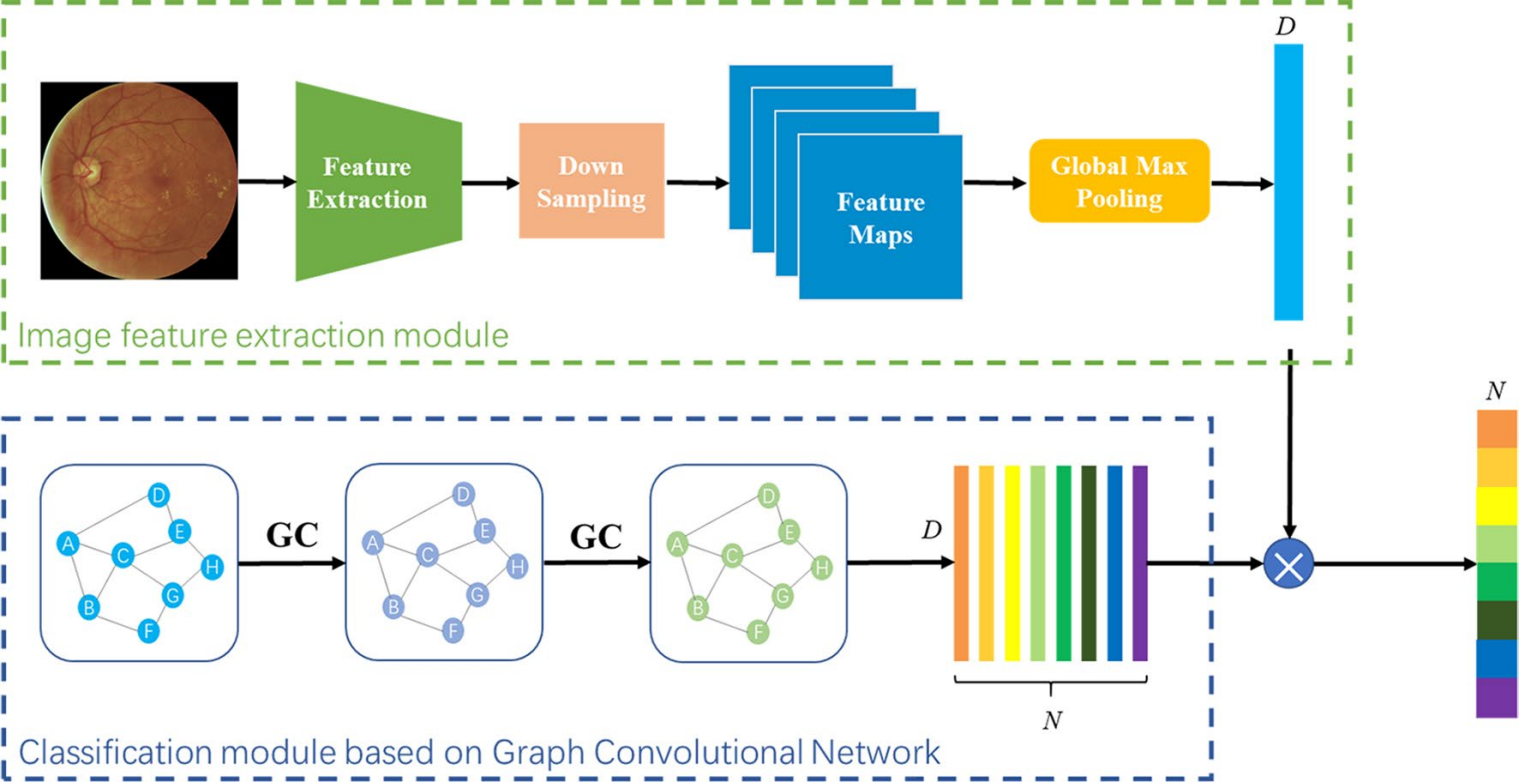
Multi-label classification model of fundus images based on GCN

We adjusted and improved ML-GCN proposed by chen’s team [19] to construct a multi-label classification model which would be suitable for fundus images. The model was composed of two parts: image feature extraction module and GCN-based classification module. The model frame is shown in Fig. 1, where $N=8$ and $D=2048$.

**Fig. 2:**
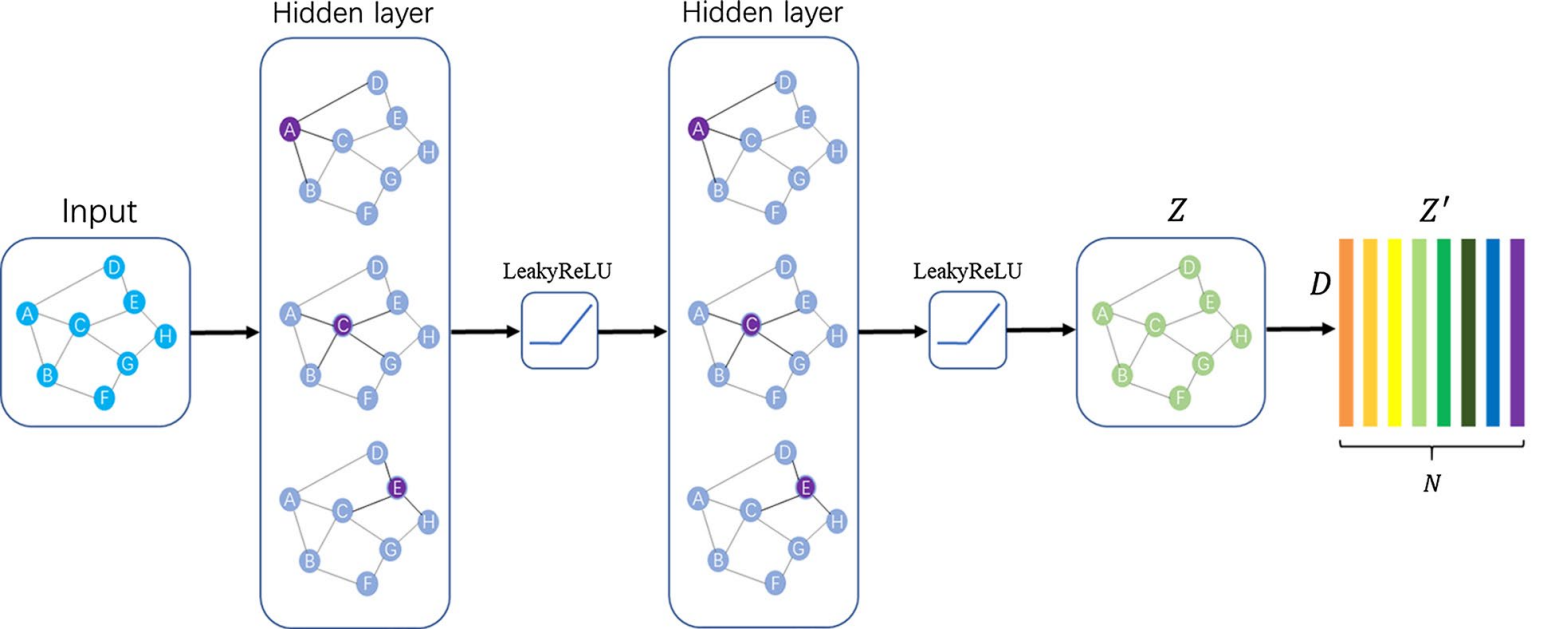
The GCN architecture diagram

#### Image feature extraction module

The image feature extraction module used a CNN-based model to extract fundus image features. In our experiments, we tested different CNN models (VggNet [20], ResNet [21], DenseNet [22]), and finally decided to use ResNet-101 [21] to extract lesion features. Considering that some lesion features (microaneurysms, hard exudations, soft exudations, etc.) would be greatly difficult to recognize at low resolution, we used the $1024\times 1024$ size fundus images as the input of ResNet-101. At this point we could get $2048\times 32\times 32$ feature maps from the “conv5_x” layer of ResNet-101, and then we used two convolution layers to downsample the feature maps, and the layers’ stride is 2, kernel size is $3\times 3$. Finally, we used the “adaptive max-pooling” to get the one-dimensional image features $F\in {\mathbb{R}}^D$, where ${\hbox{D}}=2048$.

#### GCN-based classification module

We used the GCN-based classification module to build classifiers by modeling label dependencies. GCN network is a kind of neural network that performs operations on graphs to learn a classification function [23, 24].

The GCN architecture used in this article is shown in Fig. 2. The input of GCN consists of two parts:Feature matrix $X\in {\mathbb{R}}^{N*d}$ and Adjacency matrix $A\in {\mathbb{R}}^{N*N}$.*X* is used to describe the characteristics of nodes, and *A* is a representative description of the graph structure, *N* is the number of categories, *d* is the number of features of the nodes.

**Fig. 3:**
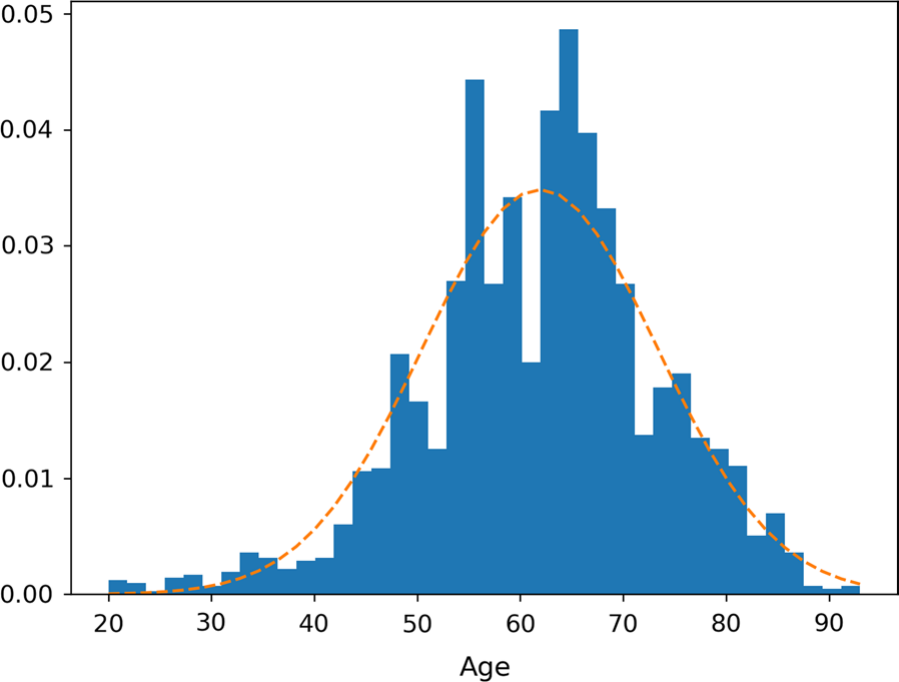
Statistics of patients age distribution

**Fig. 4:**
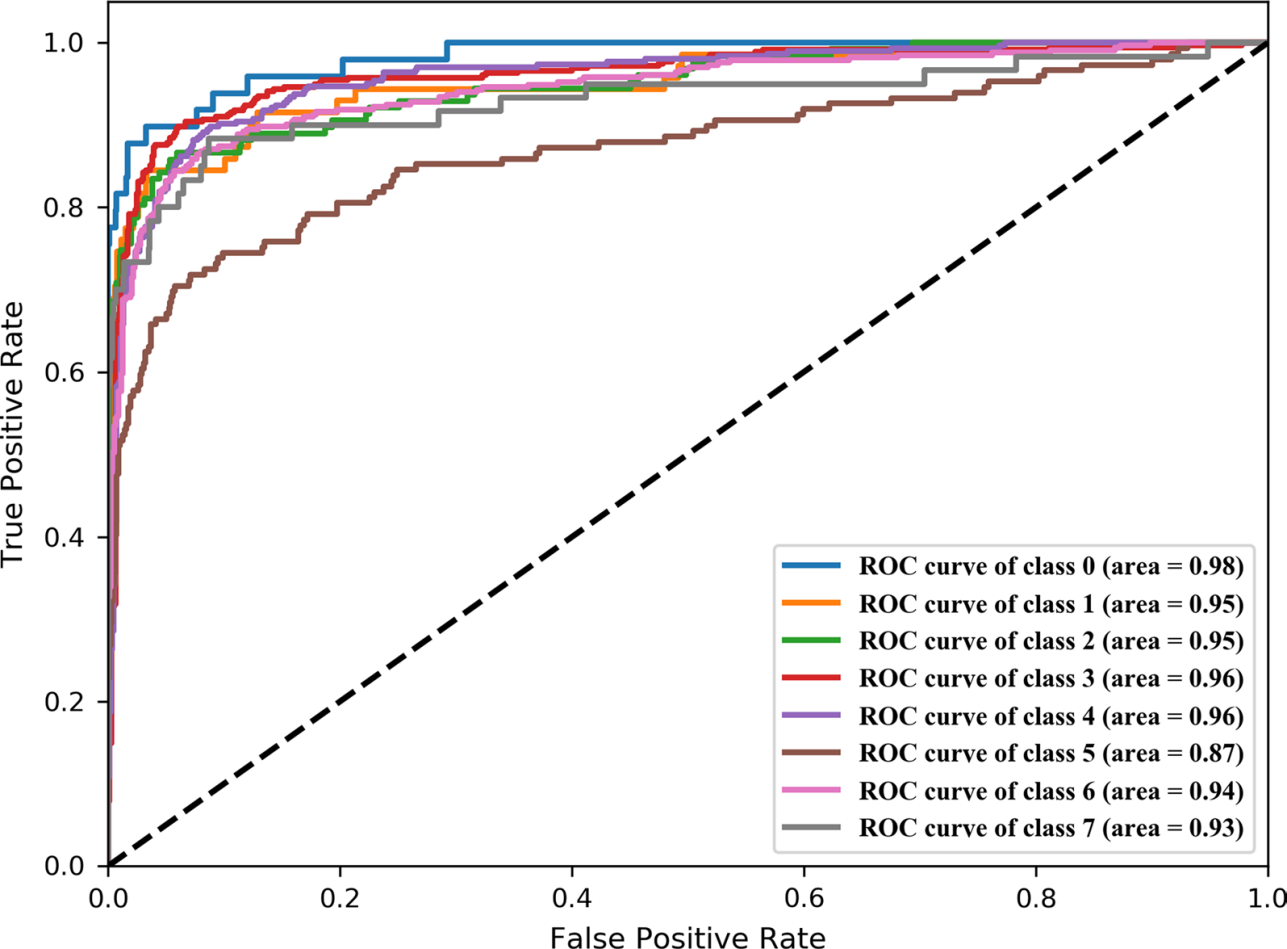
The AUC curve of each label of the model based on *X*

Every hidden layer of GCN can be expressed as:

1 $$\begin{aligned} H^{l+1}&= f(H^l,A) \end{aligned}$$

where $H^l\in {\mathbb{R}}^{N*d^l}$ is the graph-level outputs of the lth layer, $d^l$ indicates the dimensionality of node features, $H^0$ is Feature matrix *X*, *A* is Adjacency matrix. Based on the propagation rule introduced in Kipf et al. [24], $f(\cdot )$ can be expressed as:

2 $$\begin{aligned} f\left( H^l,A\right) =\sigma (\hat{A}H^lW^l) \end{aligned}$$

where $\hat{A}\in {\mathbb{R}}^{N*N}$ is the normalized version of correlation matrix *A*, $W^l$ is a weight matrix for the lth neural network layer, $\sigma (\cdot )$ is a non-linear activation function, In this experiment, we used LeakyReLU [25] as the activation function.

For the last layer, the output of GCN is $Z\in {\mathbb{R}}^{N*D}$, $Z^\prime \in {\mathbb{R}}^{D*N}$ is obtained by *Z* transpose, *D* is the feature dimension of the final node, $D=2048$. Finally, we apply the label features learned through GCN as a classifier to the image features, then we can obtain the predicted scores $y_{pred}\in {\mathbb{R}}^N$ as:

3 $$\begin{aligned} y_{pred}=FZ'. \end{aligned}$$

#### The design of the GCN input matrix

In order to explore the complex topology between lesion labels, we used the GCN network to model label dependences. It can be seen from the above that the input of GCN is composed of feature matrix and adjacent matrix. We built the feature matrix based on the word embeddings of the labels, and built the adjacent matrix based on the co-occurrence pattern of the labels in the dataset. However, since it was difficult to obtain the word embeddings of the highly medical professional lesion labels based on a universal corpus, we created a professional fundus lesion-related corpus to obtain the word vectors of lesion labels.

*The construction of feature matrix* We collected articles related to fundus lesions through keywords searching from CNKI website and extracted the abstracts of these articles to construct a corpus related to fundus lesions. We have collected a total of 10,500 related documents. We then cleaned the corpus, including removing all symbols except commas and periods, replacing English abbreviations with full names, and so on. After that we segment the corpus based on the structured perceptron model [26] of HanLP [27], and the corpus after word segmentation contained Tokens 3*M*. In order to avoid the weight interference caused by stop words, we removed stop words from the corpus. After removing the stop words, the corpus contained Tokens 2*M*. In the end, we trained the Glove model [28] based on the processed corpus and generated the label word vector to construct the feature matrix $X\in {\mathbb{R}}^{N*d}$ of the fundus lesion labels, where the word vector dimension d is set to 300.

*The construction of adjacency matrix* We referred to the method proposed by Chen et al. [19] to construct the adjacency matrix based on the conditional probability $P(L_i/L_j)$ between different labels, where *L* is the category label, and $P_{ij}=P(L_i/L_j)$ refers to the appearance probability of the label $L_i$ when the label $L_j$ appears in the Training set. Due to the use of the co-occurrence patternmode of labels in the training set to construct the adjacency matrix, the adjacency matrix will not be suitable for the test set. In order to improve the generalization ability of the model, we binarized $P_{ij}$ to obtain the binarization adjacency matrix $A^\prime$. At the same time, in order to avoid the over-smoothing of the label features caused by the binarization Adjacency matrix, we re-weighted the binarization adjacency matrix to obtain the final adjacency matrix. Therefore, the adjacency matrix can be expressed as:

4 $$\begin{aligned} A^\prime _{ij}&= {\left\{ \begin{array}{ll} 0, &{}\quad P_{ij}< j\\ 1,&{}\quad P_{ij}\ge j \end{array}\right. } \end{aligned}$$

5 $$\begin{aligned} A_{ij}&= {\left\{ \begin{array}{ll} p/\sum \nolimits _{\begin{array}{c} j=1\\ {j\ne i} \end{array}}^C A^\prime _{ij},\quad &{}i\ne j\\ 1-p,\quad &{}i=j \end{array}\right. } \end{aligned}$$

where $A^\prime$ is the binarization adjacency matrix, A is the final adjacency matrix, and p is used to control the weight of the labels and its related labels. In this article, after experimental testing, we finally decided to set $\tau =0.3, p=0.25$.

### Material and experiments

#### Dataset

The dataset we used came from the major special program for collaborative innovation in health care in Guangzhou. Data were obtained from Zhujiang Hospital of Southern Medical University, Third Affiliated Hospital of Sun Yat-sen University, Eastern Guangdong Hospitals and other Grade A class 3 hospitals between 2015 and 2018. The dataset consists of 2282 patients (1283 females, 999 males) and 7459 fundus image data (1887 cases of left eye, 1966 cases of right eyes). The image size is $1962\times 1944$. The patients are 20–93 years old, with an average age of 61.9147 ($\pm \, 11.45$). The age distribution is shown in Fig. 3.

**Table 1:** Statistics of image label distribution

Label	Age	Age standard deviation	Sex ratio (*M*/*T*)	Number
Laser scars	62.73	12.62	0.37	232
Drusen	62.63	10.66	0.35	590
Cup disc ratio	57.09	15.45	0.51	632
Hemorrhages	59.52	11.48	0.44	2149
Retinal arteriosclerosis	64.48	10.95	0.43	2686
Microaneurysms	57.47	9.91	0.49	997
Hard exudates	60.91	10.74	0.41	1857
Soft exudates	59.09	10.38	0.44	467

The fundus image lesions to be labeled in this study include laser scars, drusen, cup disc ratio ($C/D>0.6$), hemorrhages, retinal arteriosclerosis, microaneurysms, hard exudates and soft exudates. Each fundus image was annotated by two professional ophthalmologists. If there is no difference in the annotations, then the criteria are determined, otherwise they are discussed until consensus is reached. The distribution of fundus image lables is shown in Table 1.

#### Experimental design

Before training the model, we divided 7459 images into training set, validation set and test set according to a proportion of 70%, 15%, and 15%. The number of each part and the label distribution information is shown in Table 2.

**Table 2:** Fundus image label distribution

	Training	Validation	Test	Total
Laser scars	139	49	44	232
Drusen	422	71	97	590
Cup disc ratio	394	127	111	632
Haemorrhages	1400	359	390	2149
Retinal arteriosclerosis	2073	304	309	2686
Microaneurysms	705	151	141	997
Hard exudates	1201	335	321	1857
Soft exudates	324	61	82	467

At the same time, in order to verify the reliability of the created feature matrix, we built the feature vector of our model based on the other two pre-trained word vectors. One of the pre-trained word vectors is the 300d vectors which use the Glove model to train on Wikipedia-2014 and Gigaword5 datasets obtained by Pennington et al. [28]. Another pre-trained word vectors is 300d Chinese Word Vectors obtained by Li et al. [29] through usingwhich used Skip-Gram model with Negative Sampling [30] trained on the Baidu Encyclopedia dataset. We constructed respectively the lesion label feature matrix $X^{en}\in {\mathbb{R}}^{N*d}$ and $X^{zh}\in {\mathbb{R}}^{N*d}$ by searching for the words contained in the English and Chinese lesion labels in the pre-trained word vectors. In addition, we also created a random matrix $X^r\in {\mathbb{R}}^{N*d}$ based on Gaussian distribution as a control to compare the performance of each feature matrix.

When training the model, we chose “stochastic gradient descent (SGD)” as the optimizer. Among the parameters of the optimizer, the momentum was 0.9, the weight attenuation coefficient was 0.0001, the learning rate for the pre-trained model ResNet-101 was 0.01, and the learning rate for other parts of the whole model was 0.1. At the same time, we chose “MultiLabelSoftMarginLoss” as the loss function of our model, which created a criterion that optimized a multi-label one-versus-all loss based on max-entropy. Its expression is as follows:

6 $$\begin{aligned} loss\left( y_{pred},y\right) =-\frac{1}{N}*\sum _{i}{y\left[ i\right] *\log \left( \frac{1}{{1+e^{-y_{pred}\left[ i\right] }}}\right) +(1-y[i])*\log \left( \frac{e^{-y_{pred}[i]}}{1+e^{-y_{pred}\left[ i\right] }}\right) } \end{aligned}$$

where $y_{pred}\in {\mathbb{R}}^{B*N}$ is the prediction results of the model, *B* is the batch size, *N* is the number of label categories, $y[i]\in [0,1]$ is the real label input by the model.

We used PyTorch (${\hbox{version}}=1.4$) to build the multi-label classification model of the fundus images proposed in this experiment. We used GPU graphics card (NVidia GeForce GTX $1080{\hbox{Ti}}*4$) on Ubuntu16.04 system for model training, verification and testing.

#### Evaluation metrics

In order to evaluate the performance of the proposed model, Four metrics are used in this study: the average overall F1 Score (OF1), the average per-class F1 Score (CF1), the per-class accuracy (Acc) and the per-class area under the ROC curve (AUC). F1 Score is an index used in statistics to measure the accuracy of classification models, which takes into account both model accuracy and recall rate. F1 Score can be regarded as an average weighting of model accuracy and recall. The value of F1 ranges from 0 to 1 and the higher the value, the better the performance of the model. AUC is an important curve to measure the classification problem. In this study, AUC was calculated to judge the performance of the model’s ability to classify each class of the lesion label. The closer the value of AUC is to 1, the better performance the model of each lesion label’s classification has.

For each image, we assigned labels with confidence greater than 0.5 to be positive, and compared them with a ground-based true-value labels. These measures do not need a fixed number of labels per image.

The calculation formula for each indicator is as follows:

7 $$\begin{aligned} OP&= \frac{\sum _{i}{TP}^i}{\sum _{i}{({TP}^i+{FP}^i)}} \end{aligned}$$

8 $$\begin{aligned} OR&= \frac{\sum _{i}{TP}^i}{\sum _{i}{{(TP}^i+{FN}^i)}} \end{aligned}$$

9 $$\begin{aligned} OF1&= 2*\frac{OP*O R}{OP+OR} \end{aligned}$$

10 $$\begin{aligned} CP&= \sum _{i}\frac{{TP}^i}{{TP}^i+{FP}^i}/N \end{aligned}$$

11 $$\begin{aligned} CR&= \sum _{i}\frac{{TP}^i}{{TP}^i+{FN}^i}/N \end{aligned}$$

12 $$\begin{aligned} CF1&= 2*\frac{CP*CR}{CP+CR} \end{aligned}$$

13 $$\begin{aligned} Acc[i]&= \frac{{TP}^i+{TN}^i}{{TP}^i+{FN}^i+{TN}^i+{FP}^i} \end{aligned}$$

where $i\in [1,\ldots ,N]$, *N* is the number of lesion labels, the true positive *i* $({TP}^i)$ indicates that the number of fundus images where the $i_{th}$ class of lesion labels should have existed in the image and was predicted by the model to exist, the false negative *i* $({FN}^i)$ indicates that the number of fundus images where the $i_{th}$ class of lesion labels should have existed in the image but was predicted by the model to not exist, the true negative *i* $({TN}^i)$ indicates that the number of fundus images where the $i_{th}$ class of lesion labels should not have existed in the image and was predicted by the model to not exist, the false positive *i* $({FP}^i)$ indicates that the number of fundus images where the $i_{th}$ class of lesion labels should not have existed in the image but was predicted by the model to exist, *OP* and *OR* are the average overall precision and recall, *CP* and *CR* are the average per-class precision and recall.

## Results and discussion

The performance results of the model training based on different feature matrices are shown in Tables 3 and 4. *X* is the feature matrix constructed in Section “The Construction of Feature Matrix” based on the fundus lesion related corpus.$X^{rd}$, $X^{en}$, and $X^{ch}$ are respectively the feature matrix constructed in Section “Experimental Design” based on the random distributions, Wikipadia 2014, and Baidu Encyclopedia.

Among them, *OF*1 and *CF*1 values of model based on $X^{rd}$ as the baseline are 0.483 and 0.389 respectively with the worst performance. *OF*1 and *OF*1 values of model based on $X^{en}$ are 0.627 and 0.570 respectively. *OF*1 and *OF*1 values of model based on $X^{ch}$ are 0.633 and 0.582 respectively. The performance of model based on $X^{ch}$ is not much different from that of model based on $X^{en}$. *OF*1 and *OF*1 values of model based on *X* are 0.808 and 0.792 respectively with the best performance. By comparing *OF*1 and *OF*1 values of different models, it can be seen that the word vector model based on a universal corpus cannot describe the labels of fundus lesions. The feature matrix constructed based on the fundus lesions related corpus can more comprehensively and accurately represent the complex co-occurrence relationship between lesion labels, which makes model based on *X* to have better performance. Figure 4 shows the AUC curve of each label of the model based on *X*.

**Table 3:** OF1 and CF1 values of the models based on different feature matrices

	Model base on X	Model base on $X^{rd}$	Model base on $X^{en}$	Model base on $X^{ch}$
*OF*1	0.808	0.483	0.627	0.633
*CF*1	0.792	0.389	0.570	0.582

**Table 4:** Acc and ROC values of the models trained based on different feature matrices

	Model base on X		Model base on $X^{rd}$		Model base on $X^{en}$		Model base on $X^{ch}$	
	Acc	ROC	Acc	ROC	Acc	ROC	Acc	ROC
Laser scars	0.986	0.982	0.954	0.911	0.972	0.914	0.974	0.963
Drusen	0.971	0.952	0.945	0.807	0.949	0.880	0.948	0.872
Cup disc ratio	0.956	0.949	0.902	0.829	0.921	0.879	0.919	0.840
Haemorrhages	0.926	0.961	0.768	0.776	0.836	0.879	0.829	0.871
Retinal arteriosclerosis	0.914	0.957	0.719	0.742	0.783	0.813	0.805	0.837
Microaneurysms	0.921	0.871	0.847	0.698	0.885	0.823	0.897	0.831
Hard exudates	0.903	0.944	0.805	0.804	0.847	0.889	0.845	0.870
Soft exudates	0.974	0.930	0.935	0.730	0.955	0.845	0.957	0.808

The Acc and ROC values of models shown that the model had a better detection results for Laser scars, Drusen and Haemorrhages, but had a poor detection ability for microaneurysms, soft exudates and hard exudates. We speculated that the reason is that microaneurysms looked just like small, red spots in the retinal capillaries [31]. It was difficult for the model to distinguish microaneurysms from the background of the fundus images, especially when the original image was reduced when input. The 2017 report of Tan et al. [16] also verified our opinions. Tan et al. proposed a ten layers CNN architecture for DR lesion segmentation, achieved a sensitivity of 0.46 for segmentation of microaneurysms. Tan’s job shown that microaneurysms were very difficult to distinguish from the surrounding background pixels. For soft exudates and hard exudates, we found that these two kinds of lesions often accompanied multiple other fundus lesions at the same time, which made the model difficult to extract the features of all fundus lesions. In 2018, lam et al. [32] used image patches to detect retinal lesion which can avoid the interference of different fundus lesions, the proposed model achieved the AUC of 0.94 and 0.95 for detection of microaneurysms and exudates. At the same time, in order to verify the superiority of the model, we also made a comparison with the multi-label classification model of the fundus lesions proposed by Pan et al. [17]. Pan trained a multi-label classification DenseNet model based on FFA images to detect NP, microaneurysms, leakages, and laser scars with AUC of 0.880, 0.980, 0.974 and 0.967. We used fundus photographs of the retina to build a multi-label classification model. Compared to FFA images, the fundus photographs of the retina were more often used for large-scale fundus disease screening, but were more difficult for accurate fundus disease detection. Our multi-labels classification model based on GCN can detect eight types of fundus lesions at the same time, and the AUC of detecting Laser scars was higher than the model proposed by Pan et al.

## Conclusions

In the actual clinical diagnosis, there would be a variety of lesions in the fundus images. Therefore, different from the existing single classification model, we proposed a multi-label classification model based on GCN. We also tested a variety of fundus photographs of the retina to make it better applied in the real medical scene. Our experimental results on the constructed multi-center clinical data set demonstrate the promising performance and broad application of the proposed model.

In summary, we proposed a multi-label classification model based on GCN, which enable the model to learn the complex topology between lesion labels. At the same time, we constructed a related to fundus lesions corpus and verified its superiority through comparison. The multi-label classification model had been trained and verified on the real datasets. It can detect 8 types of lesions such as laser scars, drusen, cup disc ratio $(C/D>0.6)$, hemorrhages, retinal arteriosclerosis, microaneurysms, hard exudates and soft exudates in fundus images very well. Therefore, our multi-label classification model can well assist ophthalmologists in the diagnosis of DR, reduce the workload of ophthalmologists in clinical practice, and improve the diagnostic efficiency of ophthalmologists.

## Data Availability

The data is collected and integrated by the company based on the Science and Technology Program of Guangzhou (No. 201604020016). Because of the company’s regulations, the data cannot be made publicly available.

## Abbreviations

Acc: Accuracy
ANN: Artificial neural network
AUC: Area under the ROC curve
CF1: Average per-class F1 score
CNKI: China national knowledge infrastructure
CNN: Convolutional neural network
CP: Average per-class precision
CR: Average per-class recall
*C*/*D*: Cup disc ratio
DR: Diabetic retinopathy
FFA: Fluorescein fundus angiography
GCN: Graph convolutional network
Glove: Global Vectors for Word Representation
OF1: Average overall F1 score
OP: Average overall precision
OR: Average overall recall
NP: Non-perfusion
NPDR: Non-proliferative diabetic retinopathy
PDR: Proliferative diabetic retinopathy
RBF: Radial basis function
SGD: Stochastic gradient descent
